# Estimating PMTCT's Impact on Heterosexual HIV Transmission: A Mathematical Modeling Analysis

**DOI:** 10.1371/journal.pone.0134271

**Published:** 2015-08-11

**Authors:** Aditya S. Khanna, Sarah T. Roberts, Susan Cassels, Roger Ying, Grace John-Stewart, Steven M. Goodreau, Jared M. Baeten, Pamela M. Murnane, Connie Celum, Ruanne V. Barnabas

**Affiliations:** 1 Department of Medicine, University of Chicago, Chicago, Illinois, United States of America; 2 Department of Global Health, University of Washington, Seattle, Washington, United States of America; 3 Department of Epidemiology, University of Washington, Seattle, Washington, United States of America; 4 Department of Medicine, University of Washington, Seattle, Washington, United States of America; 5 Department of Pediatrics, University of Washington, Seattle, Washington, United States of America; 6 Department of Anthropology, University of Washington, Seattle, Washington, United States of America; 7 Department of Geography, University of California, Santa Barbara, California, United States of America; 8 College of Physicians and Surgeons, Columbia University, New York, New York, United States of America; 9 Fred Hutchinson Cancer Research Center, Seattle, Washington, United States of America; University of Kwazulu-Natal, SOUTH AFRICA

## Abstract

**Introduction:**

Prevention of mother-to-child HIV transmission (PMTCT) strategies include combined short-course antiretrovirals during pregnancy (Option A), triple-drug antiretroviral treament (ART) during pregnancy and breastfeeding (Option B), or lifelong ART (Option B+). The WHO also recommends ART for HIV treatment and prevention of sexual transmission of HIV. The impact of PMTCT strategies on prevention of sexual HIV transmission of HIV is not known. We estimated the population-level impact of PMTCT interventions on heterosexual HIV transmission in southwestern Uganda and KwaZulu-Natal, South Africa, two regions with different HIV prevalence and fertility rates.

**Materials and Methods:**

We constructed and validated dynamic, stochastic, network-based HIV transmission models for each region. PMTCT Options A, B, and B+ were simulated over ten years under three scenarios: 1) current ART and PMTCT coverage, 2) current ART and high PMTCT coverage, and 3) high ART and PMTCT coverage. We compared adult HIV incidence after ten years of each intervention to Option A (and current ART) at current coverage.

**Results:**

At current coverage, Options B and B+ reduced heterosexual HIV incidence by about 5% and 15%, respectively, in both countries. With current ART and high PMTCT coverage, Option B+ reduced HIV incidence by 35% in Uganda and 19% in South Africa, while Option B had smaller, but meaningful, reductions. The greatest reductions in HIV incidence were achieved with high ART and PMTCT coverage. In this scenario, all PMTCT strategies yielded similar results.

**Discussion:**

Implementation of Options B/B+ reduces adult HIV incidence, with greater effect (relative to Option A at current levels) in Uganda than South Africa. These results are likely driven by Uganda’s higher fertility rates.

## Introduction

The 2013 World Health Organization (WHO) guidelines to prevent mother-to-child transmission (PMTCT) of HIV recommend triple-drug antiretroviral treatment (ART) for all pregnant women for the duration of pregnancy and breastfeeding (Option B) or for life (Option B+) [1]. Previous guidelines recommended Option B or Option A, a short-course regimen of zidovudine (AZT) during pregnancy, single dose nevaripine (NVP) during labor, AZT plus lamivudine for seven days postpartum, and infant NVP during breastfeeding [2]. In all cases, pregnant women eligible for combination antiretroviral therapy (ART) by national treatment guidelines should receive lifelong ART for their own health [1,2].

Option B provides significant clinical and programmatic advantages over Option A because it utilizes a single regimen for both treatment and PMTCT [1]. Option B+ has the potential to simplify Option B by keeping pregnant women on treatment for life, rather than requiring repeated initiation and cessation of treatment for multiple pregnancies [3]. Although Option B+ may be more effective than Option B for PMTCT, the extent to which this is so will depend on factors such as fertility rates, breastfeeding duration, and retention on treatment, and current evidence remains inconclusive [1]. ART reduces the risk of sexual HIV transmission through viral suppression [4], hence, Options B and B+ also have the potential to decrease HIV incidence due to sexual transmission.

Prior work has shown that pregnancy is associated with increased genital HIV shedding [5–8], and an increased risk of sexual HIV transmission [aHR = 2.47; 95% CI 1.26,4.85] within HIV serodiscordant partnerships [9]. PMTCT interventions target women during this period of increased transmissibility, and hence may have a disproportionate impact on HIV incidence. Additionally, pregnant women are a target group that is already involved in care, and there is room to scale up coverage of PMTCT to expand the possible benefits of the treatment-as-prevention potential of PMTCT. However, little is known about the population-level effects of Options B or B+; a full assessment requires a detailed analysis of impact on sexual transmission of HIV. Two unanswered questions are: How do Options B and B+ impact adult HIV incidence, at different levels of coverage? Is there a substantial reduction in adult HIV incidence under Option B+, given that it may be simpler but costlier to implement? Previously, an ethnographic study in Tanzania showed that women preferred Option B due to its shorter treatment duration [10], and a clinical trial studying benefits of Option B+ is in progress in Malawi [11]. Two modeling studies on the benefits of WHO PMTCT guidelines focused exclusively on outcomes related to mother-to-child transmission [12,13], and one other considered sexual transmissions in addition to MTCT [14]. Option B+ was found to have the largest improvement for maternal and infant life expectancy in Zimbabwe [13], and was also the most cost-effective in a comparative analysis of Kenya, Zimbabwe, South Africa and Vietnam [14]. However, none of the prior modeling studies explicitly compared different PMTCT regimens at different coverage levels, or used partnership-level data and the sexual networks through which HIV infections transmit. While Gopalappa et al. [14] did consider adult HIV infections, they relied on an estimate of the number of infections averted per person-year on ART rather than modeling sexual transmission of HIV directly.

Thus, while some work on the effects of mother-to-child transmission has been undertaken, very little is known about the impact of PMTCT on adult HIV incidence, in particular the amount by which different PMTCT regimens may reduce horizontal transmission, and how these reductions vary with PMTCT coverage. We analyze the impact of PMTCT regimens exclusively on adult HIV incidence, and consider the impact on incidence at different coverage levels, while focusing explicitly on fertility rates as a determinant of this impact.

We hypothesized that Option B+ substantially decreases HIV incidence in adults due to sexual transmission compared to Option B, which decreases HIV incidence more than Option A, and that the magnitude of this decrease correlates with fertility rates. While it seems likely that Options B and B+ will each have a greater effect on HIV incidence, we have little intuition on *how much* greater the effect will be, especially when coverage levels and fertility rates are incorporated in the model. These hypotheses were tested in two sub-Saharan African regions: southwestern (SW) Uganda, with moderate HIV prevalence (about 10% [15]) and a high fertility rate (total fertility rate of 5.9 per woman [16]), and KwaZulu-Natal (KZN), South Africa, with a high HIV prevalence (about 25% [17]) and lower fertility rate (total fertility rate of 2.2 per woman [16]). Mathematical models were used to estimate the impact of Options A, B, and B+ on HIV incidence due to heterosexual transmission at different coverage levels in each settings. In scenario analyses, we assessed the impact of fertility rates on our results, and their sensitivity to ART initiation at a higher CD4 count.

## Materials and Methods

Dynamic, stochastic, network-based models simulated HIV transmission among persons aged 18–55 years in SW Uganda and KZN, and were parameterized using demographic, biological, behavioral, and treatment data from those settings. Methods are summarized here and provided in full detail in the Supplementary Appendix. The models were derived from the exponential-family random graph modeling (ERGM) framework, and programmed using the Statnet [18] packages in the R programming language. In all models, one simulated timestep was defined to be 14 days.

**Fig 1:**
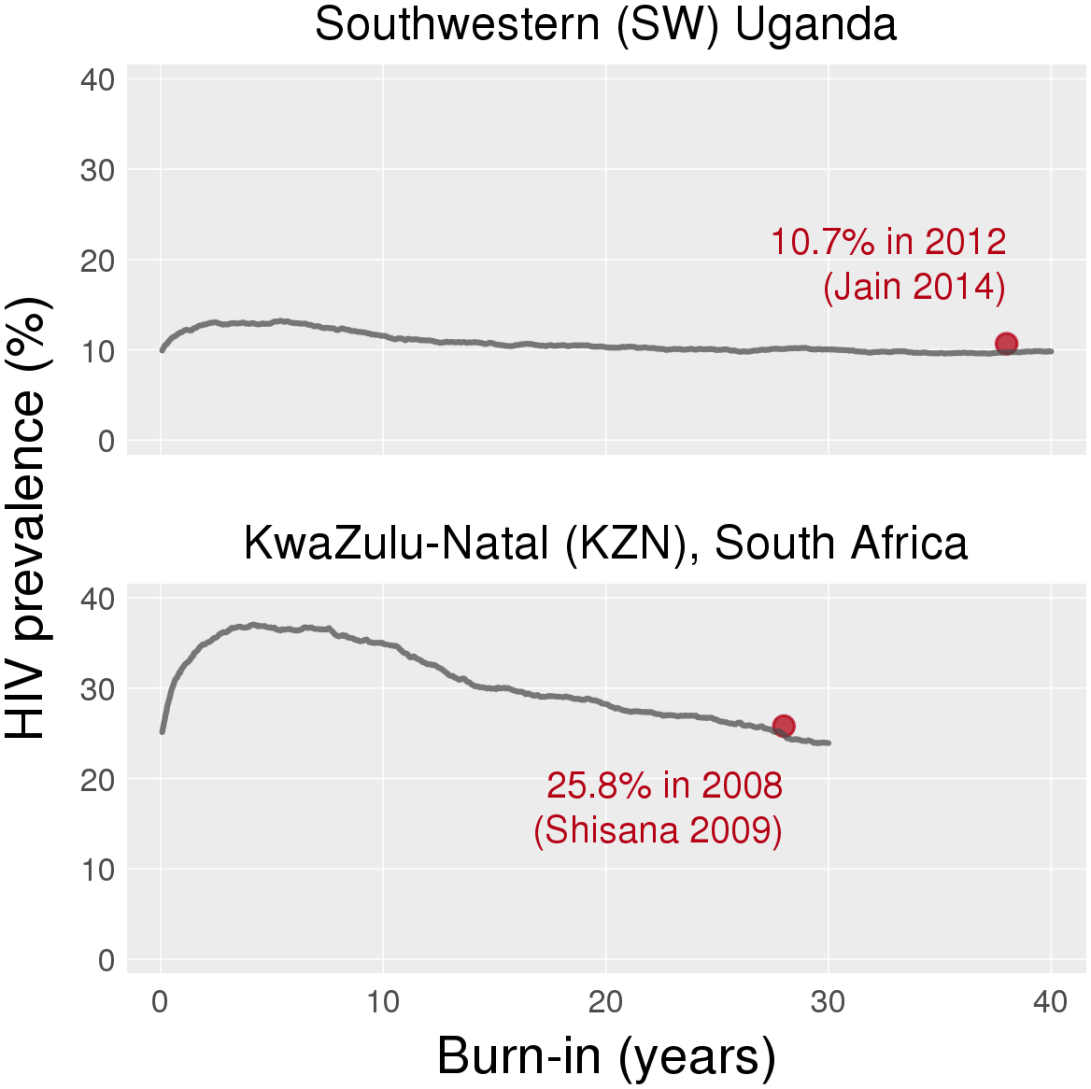
Prevalence plots to produce baseline epidemics in Uganda and South Africa.

Baseline HIV epidemics were simulated to capture existing epidemic features in the two countries, including ART coverage levels and Option A for PMTCT. (For the rest of this paper, the term “ART” denotes combination antiretroviral treatment that is initiated not as part of a PMTCT regimen.) The baseline simulation ran for 40 years in SW Uganda and 30 years in KZN to achieve approximately stable HIV incidence and prevalence (Fig 1). These baseline models were created to capture the late-stage HIV epidemics in Uganda [19] and South Africa [20], and the current rates of new infections in both countries. While both countries show signs of declining incidence, it is important to remember that both ART and PMTCT have been scaled up in the recent past; calibration of our baseline models did not include ART or PMTCT scale-up, and we did not aim to model these trends. Our goal was to obtain reasonable epidemic outcomes (incidence [21,22] and prevalence [15,17]) that are consistent with estimates from other published studies, to serve as the starting point for modeling interventions. The baseline models should not be interpreted as a reconstruction of the historical trajectory of the epidemic.

After the baseline phase, PMTCT interventions Options A, B, and B+ were simulated over ten years. Our baseline and intervention simulations only included heterosexual transmission; we did not include the impact of treatment on mother-to-child transmission (MTCT) in our models. Our primary question was to consider changes in heterosexual incidence rates over 10 years, and our modeled population included individuals between 18 and 55 years of age. Because the interventions were only modeled over 10 years, any children born during this time would not age enough to join the sexually active model population. Therefore, including transmission to newborn infants would not directly impact outcomes. However, we did include HIV prevalence at entry consistent with data for infection rates among 18-year old men and women (on average, about 3% for both Uganda [23] and South Africa [17]).

In Option A, HIV-infected pregnant women with CD4>350 cells/μl received AZT from the first antenatal visit until delivery [2]. In Option B, HIV-infected pregnant women with CD4>350 cells/μl received triple-drug PMTCT from the first antenatal visit until the conclusion of breast-feeding at 12 months postpartum [2]. In Options A and B, pregnant women with CD4≤350 cells/μl initiated lifelong ART. In Option B+, all HIV-infected pregnant women initiated lifelong ART regardless of CD4 count [1].

Each PMTCT intervention was simulated at three population coverage levels: “Current ART and PMTCT”, “High PMTCT” (with ART at Current levels), and “High ART and PMTCT” (Table 1). Current coverage included initiation at 22 weeks gestation, with 57% [23] of HIV-infected pregnant women accessing either PMTCT or ART (“uptake”) in Uganda and 89% [24] in South Africa. We assumed 75% adherence to PMTCT in this population [25]. Coverage was calculated as the product of uptake and adherence: 43% in SW Uganda and 67% in KZN. This method makes the conservative assumption that non-adherent individuals receive no benefit of treatment and have the same outcomes as untreated individuals.

**Table 1:** Coverage levels for the three scenarios that we modeled. Parameter estimates for Southwestersn Uganda (SW Uganda) and KwaZulu-Natal (KZN) are separated by commas.

	Coverage Level		
	Current	High PMTCT	High ART and PMTCT
**PMTCT**			
PMTCT initiation (weeks)	22, 22	14 [62], 14 [62]	Same as High PMTCT
PMTCT uptake (%)	57 [23], 53 [24]	90, 90 [assumption]	Same as High PMTCT
**ART**			
CD4 count at ART initiation (cells/μl)	131 [26], 100 [63]	131 [26], 100 [26]	350, 350 [assumption]
ART uptake (%)	48 [64], 53 [64]	48 [64], 53 [64]	90, 90 [assumption]
Proportion of those on ART who are virally suppressed (%)	88 [64], 85 [64]	88 [64], 85 [64]	Same as Current

Current coverage assumed ART initiation for males and non-pregnant females at a CD4 count of 131 cells/μl [26] at 48% uptake in Uganda, and 100 cells/μl [27] at 53% uptake in South Africa. Adherence was estimated by the proportion of individuals on treatment who were virally suppressed: 88% in SW Uganda and 85% in KZN [28]. As with PMTCT, ART coverage was the product of uptake and adherence: 43% in Uganda and 45% in South Africa. These coverage levels determine the proportion of HIV-infected individuals who will ever access ART in the baseline model.

We defined High PMTCT coverage as PMTCT initiation at 14 weeks gestation [1], with 90% uptake in both regions. Adherence remained at 75%. In KZN, the main change in this scenario was earlier initiation of PMTCT, because uptake was already close to 90%. In Uganda, the High PMTCT scenario included substantive changes to uptake and timing of PMTCT initiation.

Under High ART and PMTCT coverage, the High PMTCT settings were retained and ART uptake for men and non-pregnant women was set to 90% in both regions, with initiation at CD4≤350 cells/μl). ART adherence levels did not change [28]. All interventions are rolled out in the first year of the intervention at the specified levels.

### Data

Our primary data source was a prospective study of home HIV testing and counseling (HTC) to improve testing and linkage to care [28]. Home HTC was offered to consenting adults (≥18 years) in defined geographic regions in the Mbarara district of SW Uganda, and Vulindlela district of KZN, South Africa. A total of 2,121 individuals in Uganda and 1,272 individuals in South Africa were tested. The study also included a pre-intervention community survey (n = 232 in SW Uganda and n = 268 in KZN) [28]. The study provided model parameters for sexual network characteristics, including the momentary (cross-sectional) distribution of the number of partnerships, start and end dates of the last three partnerships, mixing by age, and ART uptake and adherence. The sexual network data from the empirical study represented longer-term primary and casual partnerships. Due to limitations of this data, we did not model short-term or once-off sexual partnerships. Published sources were used to parameterize additional components of our models, as described in the Supplementary Appendix.

### Components of Simulation

Each country was populated with 5,000 individuals at the start of the simulations. At each timestep, our simulations contained the following steps, with input parameters described in Table 2:

**Table 2:** Input parameters for our models.

	SW Uganda	KZN	References
**Behavior**			
Mean partnership duration (years)	11.8	8.8*	[28]
Mean number of partnerships (per person)	0.8	1.0	[28]
Frequency of unprotected sex (per partnership per week)	2.4	2.4	[49]
Distribution of number of partnerships	Men: 0 (35%), 1 (52)%, 2 (10%), 3 (3); Women: 0 (22%), 1 (76%), 2 (2%)	Men: 0 (14%), 1 (66%), 2 (11%), 3 (5%), 4 (2.5%), 5 (2.5%); Women: 0 (24%), 1 (73%), 2 (2%),	[28]
Age mixing	Mixing matrix in appendix	Mixing matrix in appendix	[28]
**Biology**			
Duration of: Acute stage Chronic stage Late stage	135 days		[65]
	1742 days		[66]
	1424 days		[67]
Level of: Peak viremia Viral set point Max. late stage viremia	6.17 log		[68]
	4.2 log		[68]
	5.05 log		[68]
Lifespan of untreated individuals	3301 days		[67]
**Infection Transmission**			
Per act (chronic stage)	Specific to viral load (details in appendix)		[48]
Mulitipliers for:			
Acute stage	4.98		[49]
Late stage	3.49		[49]
HSV infection in either partner	2.14*population prevalence (68%)		[48]
Pregnancy of HIV-infected	1.7		[69]
Pregnancy of HIV-uninfected	2.5		[69]
Circumcision of uninfected man	0.53		[48]
**Combination ART**			
Change in CD4	CD4 count recovers by 15 cells/μl every month until pre-infection level [40] or for 3 years [38], whichever is first		Inline
Changes in viral load	Declines to 50 copies/ml in 4 months		[46]
**PMTCT**			
CD4 count during treatment	Option A: Increases 50 cells/μl from initiation to delivery; Option B: Increases 15 cells/μl every month until pre-infection level; Option B+: Increases 15 cells/μl every month until pre-infection level or 3 years, whichever is first		[40]
CD4 count upon cessation of treatment	Option A: Declines to pre-treatment levels in 1 month [40]; Option B: Declines to pre-treatment levels in 2 months; Option B+[45]: NA		Inline
Viral load during treatment	Option A: Declines 1.1 log from initiation to delivery [40]; Option B: Declines to 50 copies/ml in 4 months [45]; Option B+: Same as Option B [45]		Inline
Viral load upon cessation of treatment	Option A: Increases to pre-treatment levels in 1 month [41]; Option B: Increases to pre-treatment levels in 2 months; Option B+: NA		Inline
**Pregnancy**			
Age-specific fertility rates (per 1000 person years)***	See Table S4 in S1 Text		[35]
Eligibility for pregnancy	Age between 15 and 49 years ≥15 months since onset of last pregnancy		
HIV infection and pregnancy	HIV-infected women have a 47% lower age-specificfertility rate		[36]

* We increased the mean partnership duration by approximately 3 years to match incidence data.

** We decreased the proportion of women with 0 partners and increased the proportion of women with 1 partner by 20% to balance the total number of partnerships between men and women.

*** Annual fertility rates in UN Data increased by 15% to match empirical data on proportion of 18–49 year old pregnant women at any cross-section

1. Mortality: The model included age-specific mortality for uninfected individuals [29,30] and CD4-dependent mortality [31,32] for infected individuals. Additionally, individuals exited the model at age 55 when they were no longer a part of the population of interest.
2. Entry into population: Individuals entered the population at age 18, with entry rates selected to achieve a net national population growth rates of 3–4% per year in Uganda [33] and 1–2% per year in South Africa [34].
3. Formation and dissolution of partnerships: Only heterosexual partnerships were modeled. The process of partnership formation depended on the partners’ ages and the number of existing partnerships for each individual. The mean number of partnerships and momentary distribution of partnership numbers were parameterized from the Home HTC study [28]. All partnerships had an equal probability of dissolution per time step (i.e. lengths of partnerships were geometrically distributed), estimated using the mean duration of extant (ongoing) partnerships (Table 2).
4. Pregnancy: Women aged 18–49 years whose last pregnancy started ≥15 months ago were eligible to become pregnant. Pregnancy onset was modeled as a Bernoulli event with probabilities estimated from country- and age-specific fertility rates [35]. HIV-infected women were 47% less likely to become pregnant than HIV-uninfected women [36].
5. Update of CD4 count: All adults had a sex-specific uniform CD4 count until seroconversion (518 cells/μl in men and 570 cells/μl in women [37]). After seroconversion, CD4 counts of untreated individuals declined as a function of age and sex [37]. For individuals initiating ART or Option B+, CD4 counts increased for three years after initiation [38], or until they returned to pre-infection levels [39], whichever occurred first. Under Options A or B, CD4 counts increased for the duration of treatment [40], and declined to pre-treatment levels in one [41] or two [42] months after cessation of treatment, respectively.
6. Update of viral load: Viral load trajectories were modeled as curves [43,44] defined by: time to peak viremia; duration of acute, chronic, and late-stage infection; magnitude of peak viremia; set-point viral load; and maximum late-stage viral load. We assumed each part of this curve to be linear. For women receiving Option A, viral load declined by 1.1 log (base 10) copies/mL between treatment initiation and delivery [40]. For individuals receiving ART, or Options B or B+, viral load declined to 50 copies/mL over 4 months [45,46]. Viral load returned to pre-treatment levels in one and two months upon cessation of Options A [41] and B [47], respectively.
7. HIV transmission: The probability of HIV transmission per sex act in serodiscordant couples was a function of viral load of the infected partner [48], circumcision status of the uninfected male partner, and pregnancy status of the female partner, and was adjusted for population prevalence of herpes simplex virus type 2 (HSV-2). The probabilities of transmission per timestep were calculated using the binomial formula, assuming a constant frequency of unprotected heterosexual intercourse [49].

To obtain reasonable HIV prevalence and incidence as outputs in the model, in South Africa, we increased the mean partnership duration by approximately 3 years to match incidence data. In Uganda, to balance the number of partners reported by men and women, we decreased the proportion of women with 0 partners and increased the proportion of women with 1 partner by 20%. In both countries, annual fertility rates in UN Data were increased by 15% to match empirical data on proportion of 18–49 year old pregnant women at any cross-section.

### Outcomes

The primary outcome was adult HIV incidence in the tenth year of implementation (not cumulative over the ten years), averaged over 10 model simulations. We computed 95% confidence intervals using a theoretical *t*-distribution, defined as $\overset{-}{X}\pm \;t_{df\;=\;(n-1),\;0.975}^{*}\frac{\sigma }{\sqrt{n}}$, where *X* is the outcome of interest, *n* is the number of simulation runs, and *σ* is the standard deviation. These confidence intervals only capture variation across simulations, not variation due to changing parameter values.

A secondary outcome was the proportion of all HIV-infected individuals (including treated and untreated individuals in the denominator) who were virally suppressed (viral load ≤100 copies/mL) for all interventions and coverage levels (see Supplementary Appendix). To explore the relationship between PMTCT coverage, viral suppression, and HIV incidence, we calculated incidence rates and the proportion virally suppressed after ten years for Option B+ at Current ART coverage and PMTCT coverage of 20%, 40%, 60%, and 80%, with initation at 14 weeks gestation.

### Scenario Analyses

To understand the relative impact of age-specific fertility rates (ASFRs) as a determinant of the impact of PMTCT on HIV incidence rates, the two countries’ ASFRs were switched and the change in incidence between Options A at Current coverage and Option B+ at High PMTCT (and Current ART) coverage was estimated.

In January 2014, Ugandan HIV treatment guidelines extended ART to all HIV-infected individuals with CD4≤500 cells/μl [50]. South Africa started to implement the same policy in January 2015 [51]. We estimated the effect of PMTCT interventions with this revised ART eligibility criterion for each country. We revised the mean CD4 count at initiation to 174 cells/μl in SW Uganda, and 168 cells/μl in KZN. ART coverage for SW Uganda did not change, and was revised to 51% of all HIV-infected individuals for KZN (see Supplementary Appendix for details). We estimated incidence rates for each region (with their original ASFRs) under Option A at Current coverage, and Option B+ at Current ART and High PMTCT coverage.

## Results

For SW Uganda, the baseline model resulted in 10% HIV prevalence and an incidence of 1.00 per 100 person years (py) [95% CI: 0.94, 1.05]; these estimates compared well with observed data (Fig 1) [15,21]. Similarly, for KZN, model output for current HIV prevalence (25%) and incidence (2.52 per 100 py; [95% CI: 2.31, 2.73]) were consistent with observed estimates [17,22]. We compared incidence rates under different PMTCT interventions at different coverage levels to baseline estimates, where baseline reflects a continuation of Option A at Current coverage levels for 10 years. All mean incidence rates apply to the tenth year of intervention in units of new infections per 100 py, with the 95% CI in brackets.

**Fig 2:**
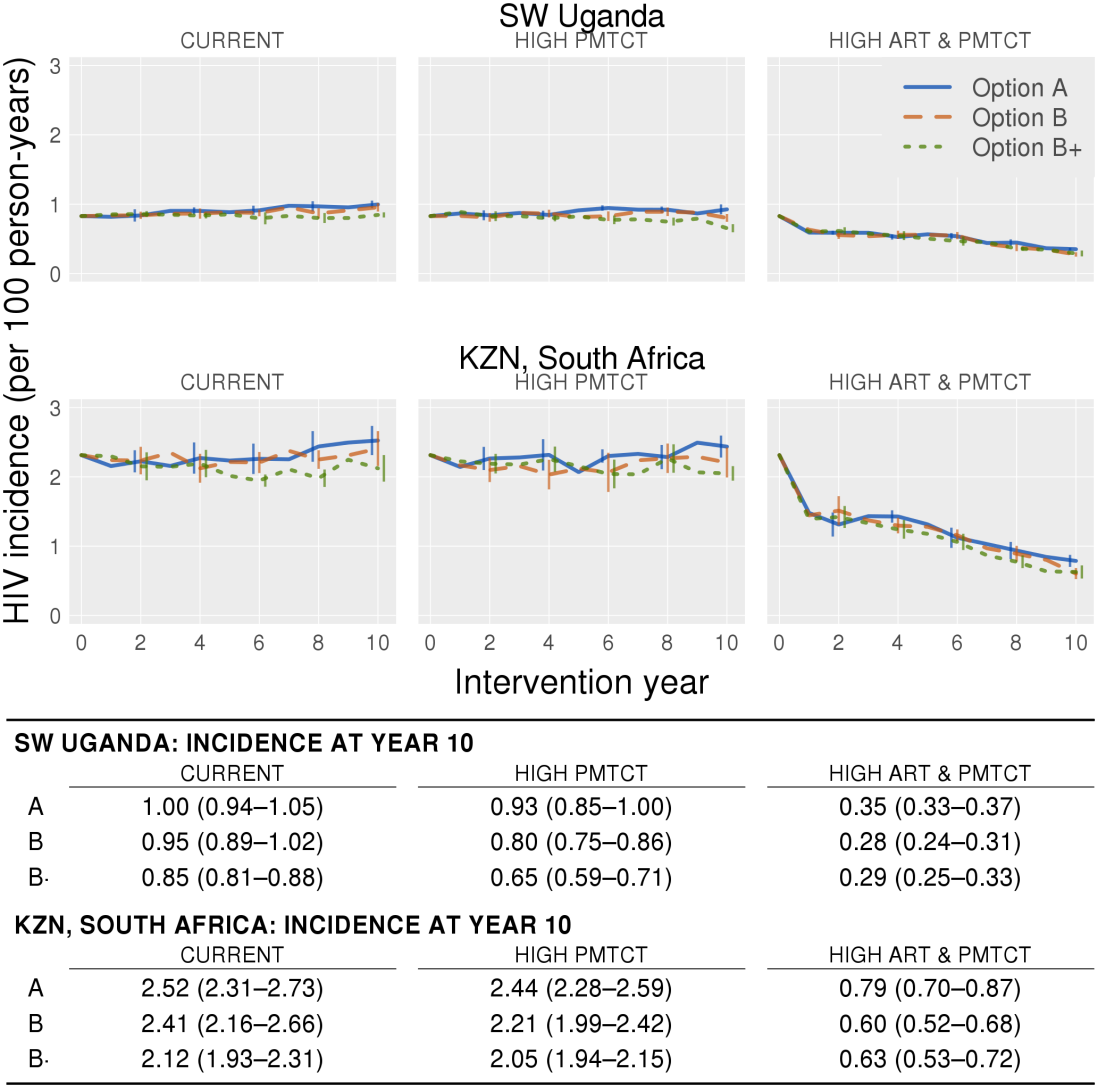
Annual incidence rates averaged over ten simulations for the ten-year intervention period in Uganda (top row) and South Africa (bottom row). Each graph shows all three PMTCT interventions; the first, second and third columns represent Current, High PMTCT and High ART and PMTCT coverage respectively. The error bars show 95% confidence intervals. The table below shows the mean incidence rate in the tenth year of the intervention, 95% confidence intervals are in the parentheses.

For SW Uganda, Options B and B+ at Current coverage produced mean incidence rates of 0.95 [0.89, 1.02] and 0.85 [0.81, 0.88] (Fig 2), corresponding to declines of 5% and 15% from baseline, respectively. With Options A, B, and B+, the High PMTCT intervention produced mean incidence rates of 0.93 [0.85, 1.00], 0.80 [0.75, 0.86] and 0.65 [0.59, 0.71], respectively, corresponding to declines of 7%, 20% and 35% relative to baseline. With the High ART and PMTCT intervention, Option A produced a mean incidence rate of 0.35 [0.33, 0.37] (mean decline of 65% relative to baseline), while Options B and B+ produced similar incidence rates of 0.28 [0.24, 0.31] and 0.29 [0.25, 0.33] (mean declines of 72% and 71%), respectively.

In KZN, at Current coverage, Options B and B+ produced mean incidence rates of 2.41 [2.16, 2.66] and 2.12 [1.93, 2.31], corresponding to relative declines of 4% and 16% from baseline, respectively (Fig 2). At High PMTCT coverage, Options A, B, and B+ produced mean incidence rates of 2.44 [2.28, 2.59], 2.21 [1.99, 2.42], and 2.05 [1.94, 2.15]–declines of 3%, 12% and 19% respectively. At High ART and PMTCT coverage, Options A, B, and B+ produced mean incidence rates of 0.79 [0.70, 0.87], 0.60 [0.52, 0.68] and 0.63 [0.53, 0.72], corresponding to mean declines of 69%, 76%, and 75%, respectively.

**Fig 3:**
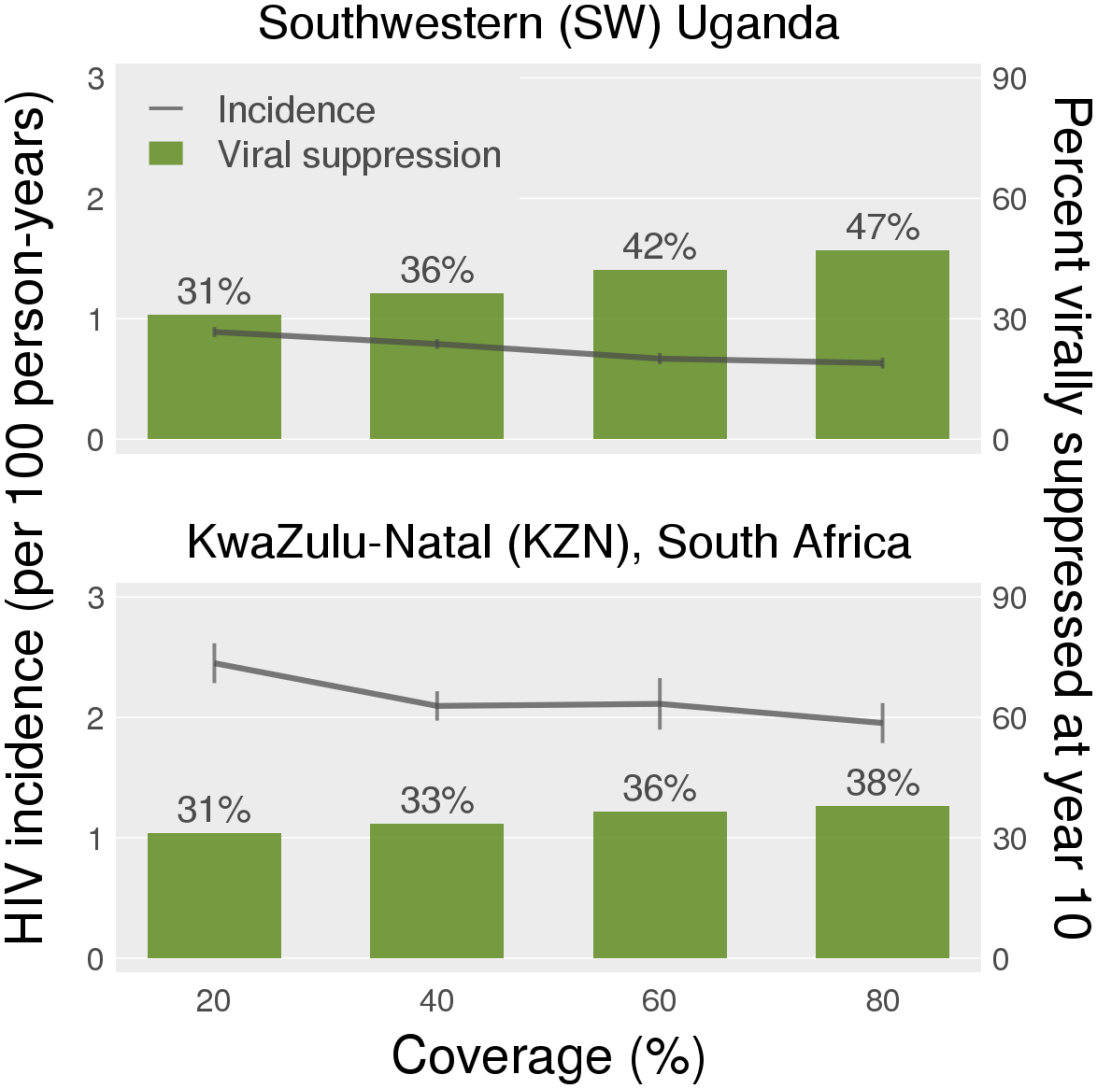
Mean HIV incidence rate in tenth year of Option B+ with High PMTCT coverage set at 20%, 40%, 60% and 80%. ART coverage is held constant at Current levels. On the right axis, we see the mean proportion of virally suppressed individuals (viral load < 100 counts/ml), at the end of the tenth year of intervention.

As coverage for Option B+ increased from 20% to 80% with ART coverage held at Current levels, the proportion of all HIV-infected individuals who were virally suppressed increased from 31% to 47% in SW Uganda, and from 31% to 38% in KZN (Fig 3). For the same coverage levels, mean incidence in SW Uganda declined from 0.89 [0.85, 0.93] to 0.63 [0.59, 0.68] (mean decline of 29%), and in KZN from 2.45 [2.28, 2.62] to 1.95 [1.79, 2.12] (mean decline of 20%).

### Scenario Analyses

In SW Uganda with South African ASFRs, mean HIV incidence was 0.99 [0.90, 1.08] in the tenth year under Option A at Current coverage, and 0.83 [0.73, 0.92] under Option B+ at High PMTCT coverage, a decline of 16%. In KZN with Ugandan ASFRs, mean incidence was 2.25 [2.06, 2.45] with Option A at Current coverage, and declined by 27% to 1.64 [1.45, 1.83] under Option B+ at High PMTCT coverage.

In SW Uganda, under revised ART eligibility criterion of CD4≤500 cells/μl, mean HIV incidence rates were 0.86 [0.77, 0.94] under Option A at Current coverage, and 0.63 [0.56, 0.69] under Option B+ at High PMTCT coverage, corresponding to a 27% decline. In KZN, under the revised criterion, mean incidence rates were 2.04 [1.89, 2.19] and 1.66 [1.51, 1.81] under the two interventions, respectively, corresponding to a 19% decline.

## Discussion

In addition to improving the health of mothers and reducing infant HIV infections, expanded coverage of PMTCT has the potential to reduce population-level adult HIV incidence in both high and low fertility settings in sub-Saharan Africa. At current levels of ART and PMTCT coverage, changing from Option A to Options B or B+ could reduce HIV incidence by similar percentages in SW Uganda and KZN (approximately 5% and 15% for Option B and Option B+, respectively). In models with increased PMTCT coverage at maintained ART coverage (High PMTCT, Current ART model), Option B+ had a substantial impact in Uganda, reducing HIV incidence by 35%, while Option B reduced incidence by 20%. In KZN, where both HIV prevalence and PMTCT coverage are high, the incidence reductions attributable to Options B and B+ were not substantially changed by improving PMTCT coverage. As expected, the largest declines in incidence in both countries were associated with higher overall ART coverage in treatment programs among adults (High PMTCT, High ART model). In this scenario, the incremental benefit of both Options B and B+ were reduced. Our conclusions are consistent with prior modeling work on the population-level benefits of early ART initiation for HIV prevention [52–55].

Our secondary analyses suggest that the variation in the findings between the two regions is attributable to the proportion virally suppressed and to fertility rates. We found a direct relationship between Option B+ coverage, the proportion of virally suppressed HIV-infected individuals, and reduction in HIV incidence. However, as Option B+ coverage increased, there was a greater increase in the proportion of HIV-infected individuals achieving viral suppression in SW Uganda than KZN, likely because the higher fertility rates in SW Uganda allowed PMTCT interventions to cover more women, and/or provide coverage for a longer period. Option B+ had a reduced impact on population HIV incidence in SW Uganda when ASFRs were lowered to South African levels, and an increased impact in KZN when ASFRs were raised to Ugandan levels. The higher mean fertility rates are correlated with a) a greater proportion of of women who are pregnant and b) a lower age at first pregnancy. Both these factors increasing the coverage of Option B+, and thus magnify its effect.

With ART eligibility at CD4≤500 cells/μl the incremental benefits of PMTCT interventions were reduced in SW Uganda. In KZN, at all coverage levels, Options B and B+ had the same impact on adult HIV incidence under the new ART eligibility criteria as under the original criteria of CD4≤350 cells/μl. In Uganda, a moderate HIV prevalence country, higher CD4 at initiation of ART may offset the added benefit of PMTCT. In a high prevalence setting such as South Africa, however, the benefit of PMTCT is retained with expanded ART coverage. Note that if ART is initiated at CD4≤500 cells/μl, Option B+ still has a greater relative impact on HIV incidence in Uganda than in South Africa.

This study has several important limitations. Self-reported partnership data had to be adjusted to balance the partnership numbers reported by men and women, and social desirability may have limited reporting of multiple partners. We only modeled long-term partnerships and did not consider casual sexual contacts because of the available data. Not including subpopulations such as commercial sex workers may have led to an overestimation of the prevention potential of PMTCT. We also assumed that all partnerships were homogeneous and equally likely to dissolve; future empirical and modeling work may consider the typologies of these partnerships, and their dissolution rates. Some model parameters were based on older data; updated parameters could influence model conclusions. The model did not reflect the dependencies between unprotected sex and risk of pregnancy. However, it did capture increased risks of HIV acquisition and transmission during pregnancy. In addition, we did not take into account certain factors that could affect HIV transmission and ART effectiveness, such as migration and mobility patterns of individuals [56], dry sex [57], HIV subtypes [58], and drug resistance [59].

The model did not consider the process of hemodilution [49], in which CD4 counts in HIV-infected pregnant women are lower than in HIV-infected, non-pregnant women, after adjusting for time of infection. Pregnant women may thus become eligible for ART sooner, and the incidence reduction associated with PMTCT may be overestimated here. Also, a high proportion of individuals on ART achieved viral suppression according to our empirical data; the impact of Option B+ on HIV incidence might be lower in other settings. We computed ART coverage as a product of uptake and adherence, equivalent to assuming that individuals who do not adhere to treatment never initiate ART. In reality, partial treatments may have some population-level effects, which implies that the effects of interventions in our study may be underestimated. Conversely, although our parameterization of PMTCT adherence does combine estimates for antepartum and postpartum adherence, we did not model the two periods separately, or account for changes in adherence after cessation of breastfeeding for women on Option B+. Since recent studies report low adherence among women in this group, we may overestimate the true impact of Option B+ [60,61]. Finally, we did not account for program costs, or conduct a full cost-effectiveness analysis. As policymakers assess which strategies are most efficient, an explicit consideration of costs is essential. We hope that our models (which are publicly available) will provide the groundwork for future work in accounting for costs.

When choosing between PMTCT regimens and selecting coverage targets, policy-makers will take into account estimates from many empirical studies and mathematical models on the efficacy and cost-effectiveness of different regimens for preventing vertical transmission. Yet in addition to those considerations, there are biological and epidemiological reasons for strengthening PMTCT programs beyond the benefits to the mother and her infant: pregnant women are at increased risk of acquiring and transmitting HIV, and high coverage of antenatal care provides good opportunities to link them to ART. Due to resource constraints, countries may have to choose between implementing Option B+ or expanding coverage under Option B, and our model results may help inform those decisions. The size of the effect of each regimen depends on PMTCT coverage, fertility rates, and coverage of ART for treatment. The difference between Options B and B+ is minimal when ART coverage is high, and is more pronounced at lower coverage. Overall, increasing Option B coverage or implementing Option B+ at current coverage both have potential to reduce HIV incidence, especially in Uganda, where fertility is high and PMTCT coverage can be improved. In South Africa, larger reductions may be achieved by expanding ART coverage to all HIV-infected persons instead of implementing Option B+.

By considering our findings on the impact of PMTCT regimens on heterosexual transmission, in addition to evidence on vertical transmission, policy makers can thus derive more realistic estimates of each regimen’s impact on overall HIV incidence. We hope that this work will provide motivation to policymakers to consider PMTCT as not just a strategy to reduce new infections in infants, but also as a strategy to reduce adult HIV incidence.

## Supporting Information

S1 Fig Proportion of infected individuals who are virally suppressed (viral count < 100 counts/ml) at the end of the ten-year simulation period in Uganda (top row) and South Africa (bottom row). Blue, orange and green bars show Options A, B, and B+, respectively.
(TIFF)

S1 Text Additional methodological information: derivation of parameters, estimation of network models, and comparison of simulated output to empirical data. (DOCX)
