# Supplementary figures and images for: Estimating PMTCT's Impact on Heterosexual HIV Transmission: A Mathematical Modeling Analysis
Source: PLoS One. 2015 Aug 11;10(8):e0134271. doi: 10.1371/journal.pone.0134271 (PMC4532442; doi:10.1371/journal.pone.0134271)

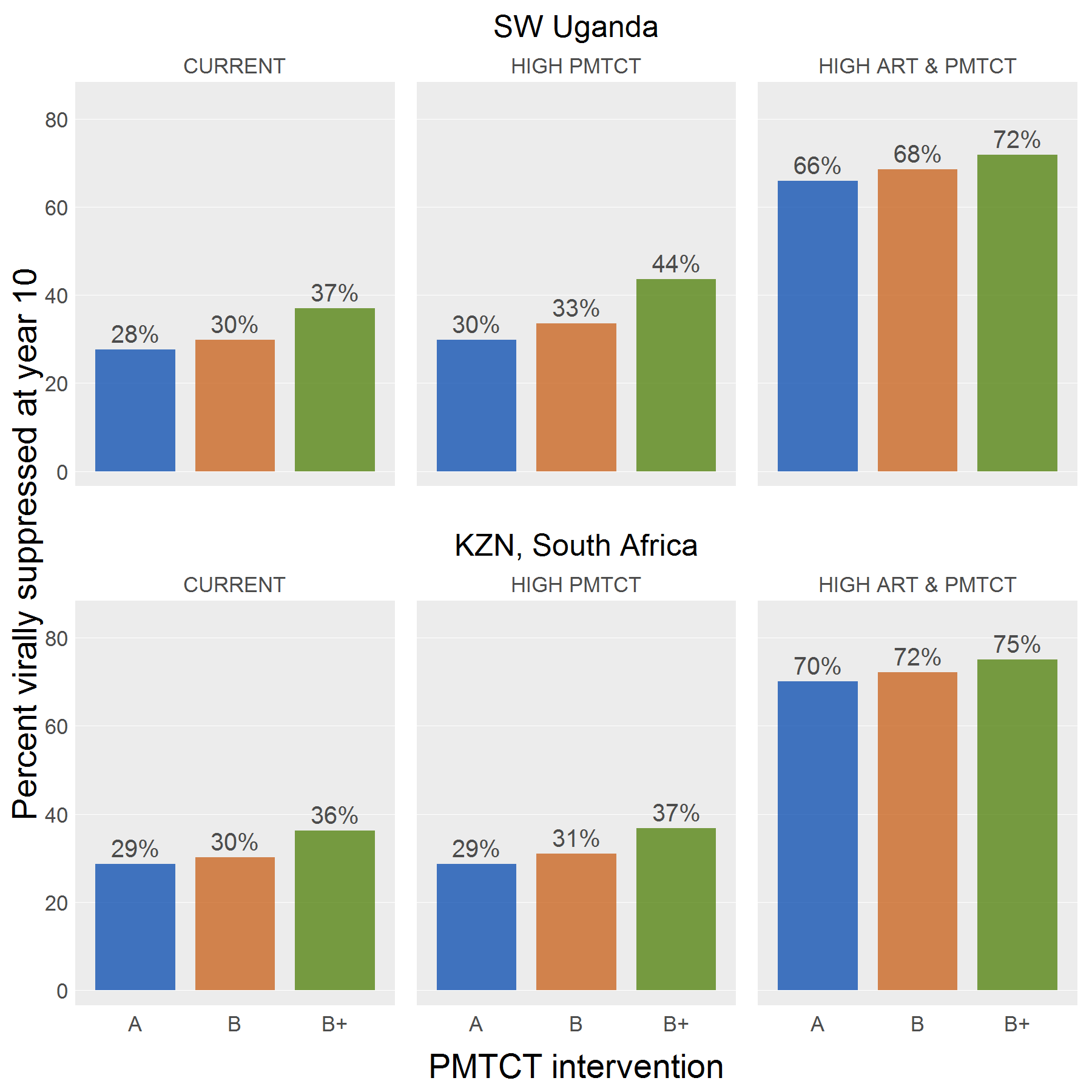

Supplement: S1 Fig — Blue, orange and green bars show Options A, B, and B+, respectively. (TIFF) [file pone.0134271.s001.tiff]
