# Supplementary material for: Estimating PMTCT's Impact on Heterosexual HIV Transmission: A Mathematical Modeling Analysis
Source: PLoS One. 2015 Aug 11;10(8):e0134271. doi: 10.1371/journal.pone.0134271 (PMC4532442; doi:10.1371/journal.pone.0134271)
Supplement: S1 Text — (DOCX) [file pone.0134271.s002.docx]

Supporting Information

Table of Contents

[1 Introduction 3](#_Toc393207106)

[2 Supplementary Methods 3](#_Toc393207107)

[2.1 Initial Population 3](#_Toc393207108)

[2.2 Models for Partnership Network 4](#_Toc393207109)

[2.3 Simulating Baseline Epidemics 7](#_Toc393207110)

[2.4 Simulating Interventions 11](#_Toc393207111)

[2.5 Estimating Parameters for ART at CD4<500 cells/µl 12](#_Toc393207112)

[3 Model Diagnostics 13](#_Toc393207113)

[3.1 Epidemic and Demographic Features 13](#_Toc393207114)

[3.2 Partnership Network Features 14](#_Toc393207115)

[4 Viral Suppression Results 16](#_Toc393207116)

[5 References 17](#_Toc393207117)

# 1 Introduction

In this Appendix, we describe several technical details that are not in the main body of the paper. We start with an exposition of the models and the populations of interest, followed by details of the network models, the various behavioral, demographic, biological, and treatment parameters, and the methods and data sources used to estimate these parameters. We end with a comparison of simulated model outputs with empirical data. Our computer programs and supporting documentation are available at: https://github.com/khanna7/Development

# 2 Supplementary Methods

Our methods followed a structure similar to prior network models of HIV transmission [1,2]. We followed the following four steps for each country [1]:

1. We generated an initial population (described in Section 2.1).
2. We estimated heterosexual partnership networks. The models for the partnership networks are described in Section 2.2.1, and the parameter estimation procedures for these networks are in Section 2.2.2.
3. We simulated baseline epidemics to capture the generalized nature of the epidemic in the two countries. It is here that the seven steps described in the main body of the paper were modeled, and the various demographic, biological, and treatment processes occured. We describe these processes, and the parameters required to model them, in Section 2.3.
4. At the conclusion of the baseline phase, we simulated PMTCT interventions at various coverage levels. Each intervention at each coverage level was simulated ten times, and we then drew conclusions about the efficacy of these interventions.

## 2.1 Initial Population

Our initial population consisted of 5000 individuals in each country. We defined the age and sex distribution in the two countries using census data [3,4]. We randomly seeded the Ugandan population with 10% HIV prevalence, and the South African population with 25% HIV prevalence at the start. The time since infection for HIV-infected members of the starting populations was uniformly distributed between 0 and the maximum lifespan of an untreated individual. The coverage and anti-retroviral tretment (ART) status of each individual was assigned in accordance with estimates from the Home HIV Tesing and Counseling (HTC) data [5]. As time evolved, these populations were set to grow at available empirical estimates available for the two countries [3,6]**.**

## 2.2 Models for Partnership Network

The theoretical framework for modeling partnership networks using the exponential random graph models (ERGMs) is described elsewhere [7], and implemented in the Statnet package [8] in the R programming language. We explain the process of adapting this framework for our models below.

Our partnership networks were modeled using bipartite graphs, forcing sexual partnerships to occur only between men and women. The cross-sectional mean number of partnerships, and the cross-sectional distribution of the number of partnerships (momentary degree distribution) for men and women, were all estimated from Home-Based HTC data [9]. The log-odds of a partnership in these models was dependent upon the relative age of the two partners.

#### 2.2.1 Model Formulation

Consider a variable (i.e. the value of the dyad at time *t*), that has the value 1 if nodes *i* and *j* have a partnership at time *t*, and is 0 otherwise. The variable represents the rest of the network excluding the tie information of *i* and *j*. This notation allowed us to describe the formation model for Uganda as:

$$\text{logit(p(}y_{ij,t}=1|Y_{ij,t-1}^{c},y_{ij,t-1}=0))=\theta\delta_{e}e+ \sum_{d_{m}=0}^{1} \theta_{d_{m}}\delta_{d_{m}}\left( \eta\left( d_{m} \right) \right)\text{ + }\sum_{d_{f}=1}^{1} \theta_{d_{f}}\delta_{d_{f}}\left( \eta(d_{f}) \right)\text{ +}$$

$$\text{ }\sum_{i=1}^{2} \theta_{m\left( a_{m_{i}},a_{f_{i}} \right)}\delta_{m\left( a_{m_{i}},a_{f_{i}} \right)}m(a_{m_{i}}, a_{f_{i}})\text{+}\sum_{a_{m=3}}^{4} \theta_{a_{m}}\delta_{a_{m}}\left( \vartheta\left( a_{m} \right) \right)\text{+}\sum_{a_{f=3}}^{4} \theta_{a_{f}}\delta_{a_{f}}\left( \vartheta\left( a_{f} \right) \right)$$

where $e$ was the number of edges; $\eta\left( d_{m} \right)$ was the number of male nodes with degree $d_{m}$, $\eta\left( d_{f} \right)$ was the number of female nodes with degree $d_{f}$; $m(a_{m_{i}}, a_{f_{i}})$ was the number of ties between males and females in age category 1 (18-25 years), age category 2 (25-35 years respectively), age category 3 (35-45 years), and age category 4 (45-55 years); $\vartheta\left( a_{m} \right)$ was the number of edges belonging to men in age category $a_{m}$, and $\vartheta\left( a_{f} \right)$ was the number of edges belonging to women in age category $a_{f}.$ All above statistics were measured at time $t.$ The $\theta^{'}s$represented the coefficients in each case.The $\delta$ functions represented “change statistics,” defined as the change in statistics associated with a toggle of the dyad $y_{ij}$ from 0 to 1. The formation model for South Africa was identical except that we specify the number of men and women with degrees 1 and 2 (i.e. $d_{m}, d_{f}\in\{1,2\}$), instead of 0 and 1, as we did for Uganda, to ensure convergence for the South African models.

The model for partnership dissolutions was a Bernoulli model of the form

$$\text{logit(p(}y_{ij,t}=1|Y_{ij,t-1}^{c}))=\theta_{\text{diss}}$$

where $\theta_{\text{diss}}$ was the coefficient associated with the dissolution of one tie (which corresponds to a change statistic of 1, in absolute value). The process of estimating the parameters for these partnership models is described in Section 2.2.2 below. We used the same dissolution model for the two countries, with the appropriate mean partnership duration for each county.

#### 2.2.2 Behavioral Parameters for Partnership Network

Our primary data source was the the Home-Based HIV Testing and Counseling Study study; any item marked “Home-Based HTC” represents data from this study [9].

1. Momentary Degree Distributions: In the Home-Based HTC sample, amongst men in Uganda, 34.8% reported 0, 52.3% reported 1, 9.8% reported 2. and 3.1% reported greater than 2 partners. Among women, 42.0%, 55.7%, and 2.3% reported 0, 1 and 2 ongoing partners respectively. Our data had sex ratios very close to 1:1, and simply applying the above data to compute the total number of partnerships reported by men and women resulted in very different estimates for total partnerships of men and women. To bring these totals closer to each other, we reduced the number of women with 0 partners by 20%, and increase the number of women with 1 partner by the same amount, to get the following partner distribution for women: 0 (22%), 1 (76%), and 2(2%). Cross-sectional distributions of number of partnerships in our South African sample were: 14.1% of men reported 0 partners, 66.1% reported 1 partner, 11.4% reported 2 partners, 4.9% reported three partners, and the rest reported 4 or 5 partners. Amongst women, 24.2% reported 0 partners, 72.5 % reported 1 partner, 1.5% reported 2 partners, and 0.2% reported greater than 2 partners.

1. Mean Number of Partnerships Per Person: We estimated a cross-sectional mean of 0.80 partnerships per person in Uganda, and 1.04 partnerships per person in South Africa. As our simulations moved forward in time, the population sizes grew at rates consistent with empirical data (more details below), and the mean number of partnerships was maintained within statistical variation of these mean values through the course of the temporal simulation.
2. Mixing Matrices by Age: We defined mixing by age in categorical counts, between non-overlapping categories of 18 to 25, 25 to 35, 35 to 45 and 45 to 55 years respectively. We present data for Uganda in Table S1, and for South Africa in Table S2.

Table S 1: Age-mixing matrix for Uganda. The table shows the proportion of all partnerships occurring between men and women in each age group

|  |  | Women | | | |
| --- | --- | --- | --- | --- | --- |
|  |  | 18-25 | 25-35 | 35-45 | 45-55 |
| Men | 18-25 | 14.9% | 1.3% | 0.0% | 0.0% |
|  | 25-35 | 15.4% | 25.8% | 1.8% | 0% |
|  | 35-45 | 1.6% | 9.6% | 11.5% | 4.2% |
|  | 45-55 | 1.5% | 2.7% | 5.5% | 4.2% |

Table S2: Age-mixing for South Africa. The table shows the proportion of all partnerships occurring between men and women of each age group.

|  |  | Women | | | |
| --- | --- | --- | --- | --- | --- |
|  |  | 18-25 | 25-35 | 35-45 | 45-55 |
| Men | 18-25 | 17.6% | 1.0% | 0.0% | 0.0% |
|  | 25-35 | 11.9% | 27.4% | 1.1% | 0.0% |
|  | 35-45 | 0.7% | 9.2% | 14.9% | 0.5% |
|  | 45-55 | 0.5% | 1.9% | 5.7% | 7.7% |

1. Mean Partnership Duration: The mean partnership age for partnerships extant on the date of the interview was 4303 days (approximately 11.8 years) in Uganda, and 2221 days (approximately 6.1 years) in South Africa. We increased this mean partnership duration in South Africa by about 3 years to obtain an incidence in the correct range; due to the simplistic assumption of an exponential distribution of the time until dissolution, this parameter estimate was the most likely to be biased. We estimated durations from the age of extant ties because our model contains a homogeneous dissolution process, which imples a geometric distribution of relation durationals, and given this assumption, the mean of extant partnerships at any given time gives an unbiased estimate of the duration [10]. However, due to this simplistic assumption for partnership dissolution , it is likely that our estimate for mean duration was biased, and therefore we increased our estimate for mean partnership by about 3 years to obtain HIV incidence in the correct range.

5. Probability for unprotected sex in partnerships: We assumed 2.4 unprotected sex acts per week, in accordance with data from Uganda [11]. In the absence of data from South Africa we assumed the same rate.

## 2.3 Simulating Baseline Epidemics

With our initial populations and partnership networks set up, we simulated our models forward in time; each time step was defined equal to 14 days. We simulated these baseline epidemics until approximately stable HIV prevalence was achieved in each of the two countries: 40 years in Uganda, and 30 years in South Africa. Each step of the simulation included the following seven processes: (1) departures, (2) recruitment into the sexually active population due to sexual maturity, (3) formation and dissolution of partnerships, (4) update of CD4 counts, (5) update of viral load, (6) update of ART status, and, (7) transmission of infection within sero-discordant partnerships. We describe estimation of the demographic, biological, and treatment parameters (Sections 2.3.1, 2.3.2 and 2.3.3) required to model these process below.

#### 2.3.1 Demographic Parameters

1. Mortality: We modeled mortality on account of the following reasons:

(a) Age of 55 years.

(b) For every HIV-uninfected individual, we modeled mortality as a Bernoulli event, and computed the daily probability of death for each individual from age-specific mortality information as presented in census data [6,12]. We computed daily probabilities per individual from annual mortality rates (per 1000).

(c) For every HIV-infected individual not on treatment, we assumed a maximum lifespan of 3301 days (approximately 9.0 years) from the time of infection [13]. At every time step prior to this lifespan, we modeled death as a Bernoulli event in both treated and untreated individuals. These mortality events were dependent on CD4 counts: for infected individuals not on treatment, we estimated probability of death from a study of South African gold miners [14], and for infected individuals on treatment, we used clinical data from South Africa [15]. We summarize data on mortality rates as a function of CD4 counts in treated and untreated individuals in Table S3.

Table S3: Mortality Rates in Untreated and Treated HIV-Infected Individuals.

| CD4 counts  (cells/µl) | Morality in untreated individuals  (per 1000 person years) [14] | Mortality in treated individuals  (per 1000 person years) [15] |
| --- | --- | --- |
| < 50 | 43.2 | 38.6 |
| 50-100 | 43.2 | 12.8 |
| 100-200 | 11.2 | 5.4 |
| 200-300 | 3.3 | 2.7 |
| 300-350 | 3.3 | 2.0 |

HIV-infected individuals not on treatment experienced mortality with a probability of one at 3301days .This maximum lifespan did not apply to individuals on treatment.

2. Recruitment: We defined recruitment as entry into our population due to sexual maturity at age 18. We modeled these recruitments as homogeneous Poisson processes to achieve average population growth rates of approximately 3-4% in Uganda [4] and 1-2% in South Africa [3].

3. Pregnancy: We assumed women between 15 and 49 years of age who were at least 15 months removed from the onset of their last pregnancy were eligible to become pregnant at any given time. Onset of pregnancy in our model was a Bernoulli event. Probability of these events were estimated from age-specific fertility rates (ASFRs) for Uganda and South Africa, as provided by UN Data (2005) [16]. We used these ASFRs, and data on age-specific HIV prevalence in the two countries (Uganda DHS [6], National Survey of South Africa [17]), to compute the ASFRs in uninfected women. We applied a 47% reduction in ferility rate for HIV-infected women, consistent with clinical data from Uganda [18]. We then calcuated the ASFRs for HIV-uninfected women in each country needed to make the weighted average across infected and unifected women match the UN data. The resulting rates (per 1000 person years) are presented in Table S4.

Table S4: Age-Specific Fertility Rates in HIV-uninfected women in Uganda and South Africa (per 1000 person years)

| Age | Uganda | South Africa |
| --- | --- | --- |
| 15-19 | 175 | 80 |
| 20-24 | 345 | 139 |
| 25-29 | 320 | 142 |
| 30-34 | 266 | 105 |
| 35-39 | 185 | 67 |
| 40-44 | 80 | 27 |
| 45-49 | 37 | 8.8 |

#### Biological and Treatment Parameters

1. CD4 trajectory: The CD4 count of uninfected men and women was assumed to be 518 cells/µl and 570 cells/µl respectively [19]. After infection, these counts were assumed to decline following mathematical relation (given by Pantazis et al. [20].)

$\text{CD4}_{t}= \beta_{0}+\beta_{1}R+\beta_{2}F+t\left( \beta_{3}+\beta_{4}R+\beta_{5}A \right)^{2}$ (1)

where $\text{CD}4_{t}$ was the CD4 count at $t$ years after sero-conversion, $\beta_{0}$ was 23.53; $R$ was an indicator of African ethnicity (set at 1 for all of our populations), with a coeffiecient $\beta_{1}$ estimated at -0.76; $F$ was an indicator for female with a coefficient$\beta_{2}$estimated at 1.11, $\beta_{3}$ estimated at -1.49 and $\beta_{4}$ estimated at 0.34; $A$ was the age at seroconversion, with coefficient $\beta_{5}$ estimated at: 0 for $15\leq A<30$, -0.1 for $30\leq A< 40$, -0.34 for $40\leq A< 50$, and -0.63 for $A\geq50$.

1. Viral load trajectory: We modeled the viral load trajectory in each infected, untreated individual as a six-parameter curve described in Table S5.

Table S5: Model of Viral Load Trajectory

| Type of Parameter | Parameter | Estimate | Source |
| --- | --- | --- | --- |
| Time | Infection to peak viremia  Infection to viral set point  Infection to late-stage infection  Infection to Death | 14 days  121 days  1877 days  3301 days | Ribeiro et al. [21]  Morrison et al. [19]  Van der Paal et al. [13]  Van der Paal et al. [13] |
| Magnitude | Level of peak viremia  Viral set point  Maximum late-stage viral load | 6.17 log  4.2 log  5.05 log | Pilcher et al. [22]  Pilcher et al. [22]  Wawer et al. [11] |

1. Treatment: We considered two types of treatment: short course ART (sc ART) for pregnant women receiving PMTCT Option A, and combination ART for pregnant women receiving Option B or Option B+ and for all patients receiving ART for their own health (i.e., CD4<350 cells/µl). Under combination ART, viral load became undetectable (50 copies/ml) after four months [23,24]. We modeled a decrease of 1.1 log in viral load in pregnant women who received Option A from the time of initiation of treatment until delivery [25]. We assumed viral load and CD4 to return to pre-treatment levels in 1 month upon cessation of Option A [26], and in two months upon cessation of Option B [24].
2. In the presence of either ART regimen, the CD4 count recovered by 15 every month (consistent with Fairall et al*.* [27]), until achieving pre-infection levels, or 3 years after initiation of treatment [28], whichever happened first.
3. Infection Transmission: We considered three stages of HIV infection: acute (onset of infection to 121 days), chronic (121 to 1878 days after infection) and late-stage (1878 days after infection to death) [13,21]. We modeled the relationship between viral load and chronic-stage infectivity (defined as the probability of transmission in any unprotected sex event), adjusted for prevalence of the Herpes Simplex Virus (HSV) in the population, in accordance with data presented by Hughes et al. [29] (Table S6).

Table S6: Viral load and Infectivity

| Viral load (log copies/ml) | Infectivity per sex act | HSV-adjusted infectivity per sex act |
| --- | --- | --- |
| 3 | 0.00028 | 0.0049 |
| 4 | 0.00082 | 0.0014 |
| 5 | 0.0024 | 0.0042 |
| 6 | 0.0068 | 0.012 |

Hughes et al. found an increase in infectivity by a factor of 2.89 for a unit increase in viral load on the log-scale. We use this result to estimate an infectivity of 0.00017 at viral load of log 2. The estimates in Table S5 show chronic-stage infectivities; we considered acute and late-stage infectivities to be increased by factors of 4.98 for the acute stage and a factor of 3.49 for the late stage [11]. Since in our models one time step equals fourteen days, we adjust these per-act probabilities to cover the 14 day period using the binomial formula, and under the assumption of 2.4 sex acts per week.

We considered a 53% reduction in transmission probability when the HIV-uninfected male partner is circumcised [29]. We also considered an increase in transmission probabilities on account of pregnancy: Infection transmission probabilities were increased by a factor of 2.5 when the infected partner was pregnant, and a factor of 1.7 when the HIV-uninfected partner was pregnant [30].

## Simulating Interventions

As discussed in the main body of the paper, at the conclusion of the baseline phase, we simulated PMTCT interventions Option B and Option B+ at current, High PMTCT and Best (Everyone) implementation levels. We also simulated a control setting with PMTCT Option A at the current level, and then expanded coverage in the High PMTCT and Best (Everyone) cases. Aside from the intervention and coverage levels, the intervention simulations followed all the same steps for the baseline scenarios explained in Section 2.3 above.

## Estimating Parameters for ART at CD4<500 cells/µl

To model the effect of PMTCT interventions when ART eligibility criteria change to CD4<500 cells/ µl, we estimated how the new criteria would affect ART coverage levels for Current ART and CD4 count at ART initiation. In South Africa, we used recent data on the CD4 distribution at enrollment into HIV care from the Hlabisa HIV Care and Treatment Programme [31]. In the home HTC study [32], 63·6% of HIV infected persons were enrolled in HIV care and of those, 71% had initiated ART. We estimated the proportion of those enrolled initiating ART within each eligible CD4 stratum (93% at CD4<=100 cells/µl, 93% at CD4 101-200 cells/µl, and 68% at CD4 201-350 cells/µl), and for reasons other than low CD4 counts (18·3%), that would be required to reach the total of 71% seen in our target population, using results from the Home HTC study for guidance [32]. We then assumed that under the new eligibility criteria, 50% of individuals enrolled in care and newly eligible would initiate ART, but that initiation rates for other persons would remain unchanged. Under these conditions, 80% of individuals enrolled in HIV care would initiate ART with the new eligibility criteria, and coverage of the entire HIV infected population would increase to 51%. In the home HTC study population in Uganda [32], nearly 100% of individuals enrolled in HIV care were already on ART, so we assumed that ART coverage would not increase with a change in eligibility criteria.

To estimate changes in CD4 count at ART initiation in South Africa, we again used data from the Hlabisa HIV Care and Treatment Programme [31] to calculate proportion of all individuals initiating ART with CD4 counts in each stratum. We then estimated the mean CD4 count at initiation within each stratum that would result in the approximately the overall CD4 count at initiation of 100 cells/ µl that we used to parameterize our model (Table 2, main text). We assumed, as above, that 50% of individuals with CD4 count 350-500 cells/µl would initiate ART under the new eligibility criteria, and that CD4 count at initiation in this group would be 380 cells/µl (toward the lower end of the range, as in the other CD4 strata). We calculated that mean CD4 count at ART initiation under new eligibility criteria would be 168 cells/µl, based on a weighted average across the strata. We followed the same process to estimate the CD4 count at ART initiation for Uganda. We used data from Geng et al. [33] to calculate proportion of all individuals initiating ART with CD4 counts in each stratum under current eligibility criteria. However, these data were not available for Uganda to determine the distribution of CD4 at ART initiation under new eligibility criteria. Therefore we used estimates from Rwanda for CD4 count at enrollment into care [34], and from South Africa for the proportion of enrolled individuals initiating ART at each CD4 count [31]. We again assumed that 50% of individuals with CD4 count 350-500 cells/µl would initiate ART under the new eligibility criteria, and we assumed that CD4 count at initiation in Uganda would be 400 cells/µl, reflecting the overall higher CD4 at initiation, compared to South Africa. We calculated that the CD4 count at ART initiation would increase to 174 cells/µl under the new eligibility criteria.

# 3 Model Diagnostics

We present a number of emergent epidemic, demographic and network features of our models, and compare them to empirical target values.

## 3.1 Epidemic and Demographic Features

Table S7: Epidemic and Demographic Features: Emergent Model Features Compared with Targets. The features are measured in all adults unless otherwise stated.

| Feature | Uganda | | | South Africa | | |
| --- | --- | --- | --- | --- | --- | --- |
|  | Model Output | Target | Citation | Model Output | Target | Citation |
| Prevalence (at conclusion of baseline) | 9.8% | ~10% | [35] | 23.9% | 22.1%-29.8% | [17] |
| Prevalence (last 10 years of baseline) | 9.8% | ~10% | [35] | 26.1% | 22.1%-29.8% | [17] |
| Incidence per 100 person years (last 10 years of baseline) | 0.82 | ~1 | [36] | 2.4 | 2.2 | [37] |
| Proportion of women who are pregnant | 13.8% | 11% | Uganda DHS calculations | 6.7% | 3.5-7.5% | [17] |
| Population growth rate (last 10 years of burnin) | 3.3% | 3-4% | [4] | 2.1% | 1-2% | [3] |

## 3.2 Partnership Network Features

Through the course of our simulations, we observed a mean of 0.8 partnerships per per person in Uganda, and 1.0 in

South Africa. We present distributions of partner numbers from our simulated networks at the last time step in Table S8 below, and age mixing matrices in our simulated networks at the last time step in Tables S9 and S10 below.

Table S8: Simulated Distributions of Number of Partnerships (at the last time step of baseline)

|  | Uganda | South Africa |
| --- | --- | --- |
| Men | Simulated: 0 (32%); 1 (50%); 2 (14%); 3 (3%)  Target: 0(34.8%), 1 (52.3%), 2 (9.8%), 3 (3.1%) | Simulated: 0 (32%); 1 (51%); 2 (14%); 3 (3%)  Target: 0 (14.1%), 1 (66.1%), 2 (11.4%), 3 (4.9%) |
| Women | Simulated: 0 (14%); 1 (81%); 2 (5%)  Target: 0 (22%), 1 (76%), 2(2%). | Simulated: 0 (17%); 1 (79%); 2 (1%); 3 (2%); 4 (1%)  Target: 0 (24.2%), 1 (72.5%), 2 (1.5%), >2 (0.2%) |

Table S9: Simulated Age-Mixing matrix in Uganda (at last time step of baseline). Numbers in parentheses represent empirical target values.

|  | | Women | | | |
| --- | --- | --- | --- | --- | --- |
|  |  | 18-25 | 25-35 | 35-45 | 45-55 |
| Men | 18-25 | 10.2%  (14.9%) | 4.3%  (1.3%) | 1.8%  (0.0%) | 1.2%  (0.0%) |
|  | 25-35 | 11.7%  (15.4%) | 20.4%  (25.8%) | 6.3%  (1.8%) | 2.4%  (0.0%) |
|  | 35-45 | 4.7%  (1.6%) | 9.9%  (9.6%) | 10.7%  (11.5%) | 3.3%  (4.2%) |
|  | 45-55 | 1.7%  (1.5%) | 2.6%  (2.7%) | 3.4%  (5.5%) | 5.2%  (4.2%) |

Table S10: Simulated Age-Mixing matrix in South Africa (at last time step of baseline). Numbers in parentheses represent empirical target values.

|  | | Women | | | |
| --- | --- | --- | --- | --- | --- |
|  |  | 18-25 | 25-35 | 35-45 | 45-55 |
| Men | 18-25 | 17.2%  (17.6%) | 6.3%  (1.0%) | 2.0%  (0.0%) | 0.3%  (0.0%) |
|  | 25-35 | 8.0%  (11.9%) | 21.4%  (27.4%) | 6.7%  (1/1%) | 1.3%  (0.0%) |
|  | 35-45 | 3.7% (0.7%) | 7.5%  (9.2%) | 12.1%  (14.9%) | 2.4%  (0.5%) |
|  | 45-55 | 1.7%  (0.5%) | 2.8%  (1.9%) | 2.6%  (5.7%) | 3.7%  (7.7%) |

# Viral Suppression Results

S1 Fig. shows the proportion of individuals who are virally suppressed for each PMTCT intervention, at each coverage level, in Uganda and South Africa. We see that with High ART and PMTCT coverage, greater than 65% of HIV-infected individuals are virally suppressed in Uganda and South Africa. However at Current coverage levels, or at High PMTCT (with Current ART), Option B+ produces a greater proportion of virally suppressed invdividuals than either Option A or Option B. This may be because with Option B+, ART does not require repeated initiation for each pregnancy. Uganda, which has a much higher age-specific fertility rates than South Africa, and likely more cases of multiple pregnancies, therefore, experiences a greater benefit of this lifelong treatment.

# References

1. Goodreau SM, Carnegie NB, Vittinghoff E, Lama JR, Sanchez J, et al. (2012) What Drives the US and Peruvian HIV Epidemics in Men Who Have Sex with Men (MSM)? PLoS One 7: e50522. doi:10.1371/journal.pone.0050522.

2. Khanna AS, Goodreau SM, Gorbach PM, Daar E, Little SJ (2014) Modeling the Impact of Post-Diagnosis Behavior Change on HIV Prevalence in Southern California Men Who Have Sex with Men (MSM). AIDS Behav 18: 1523–1531. doi:10.1007/s10461-013-0646-2.

3. Statistics South Africa (n.d.). Available: http://beta2.statssa.gov.za/.

4. United States Census Bureau (n.d.) International Database. Available: http://www.census.gov/population/international/data/idb/informationGateway.php.

5. Barnabas R V, van Rooyen H, Tumwesigye E, Murnane PM, Baeten JM, et al. (2014) Initiation of antiretroviral therapy and viral suppression after home HIV testing and counselling in KwaZulu-Natal, South Africa, and Mbarara district, Uganda: a prospective, observational intervention study. Lancet HIV 1: e68–e76. Available: http://linkinghub.elsevier.com/retrieve/pii/S2352301814700244. Accessed 3 January 2015.

6. Uganda Bureau of Statistics and ICF International (n.d.) Uganda Demographic and Health Survey 2011. Available: http://www.ubos.org/onlinefiles/uploads/ubos/UDHS/UDHS2011.pdf.

7. Krivitsky PN, Handcock MS (2014) A Separable Model for Dynamic Networks. J R Stat Soc Ser B Stat Methdology Ser B Stat Methdology 76: 29–46.

8. Handcock MS, Hunter DR, Butts CT, Goodreau SM, Morris M (2003) statnet: Software tools for the Statistical Modeling of Network Data. Available: http://statnetproject.org.

9. Barnabas R, Ying R, van Rooyen H, Murnane P, Hughes J, et al. (n.d.) No Title.

10. Krivitsky P (2009) Statistical Models for Social Network Data and Processes University of Washington.

11. Wawer MJ, Gray RH, Sewankambo NK, Serwadda D, Li XB, et al. (2005) Rates of HIV-1 transmission per coital act, by stage of HIV-1 infection, in Rakai, Uganda. J Infect Dis 191: 1403–1409.

12. Anderson B, Phillips H (n.d.) The Changing Pattern of Adult Mortality in South Africa, 1997-2005: HIV and Other Sources. Available: http://www.psc.isr.umich.edu/pubs/pdf/rr08-649.pdf.

13. Van der Paal L, Shafer LA, Todd J, Mayanja BN, Whitworth JA, et al. (2007) HIV-1 disease progression and mortality before the introduction of highly active antiretroviral therapy in rural Uganda. AIDS 21 Suppl 6: S21–S29.

14. Fielding K, Koba A, Grant AD, Charalambous S, Day J, et al. (2011) Cytomegalovirus viremia as a risk factor for mortality prior to antiretroviral therapy among HIV-infected gold miners in South Africa. PLoS One 6: e25571.

15. Lawn SD, Little F, Bekker LG, Kaplan R, Campbel E, et al. (2009) {C}hanging mortality risk associated with {C}{D}4 cell response to antiretroviral therapy in {S}outh {A}frica. AIDS 23: 335–342.

16. United Nations Statistics Division (n.d.) UN Data. A World of Information. Available: http://data.un.org/.

17. Shisana, O., Rehle T, Simbayi LC, K. Z, et al. (2008) South African national HIV prevalence, incidence, behaviour and communication survey 2008: A turning tide among teenager. Cape Town, South Africa: HSRC Press.

18. Ross A, der Paal L, Lubega R, Mayanja BN, Shafer LA, et al. (2004) HIV-1 disease progression and fertility: the incidence of recognized pregnancy and pregnancy outcome in Uganda. AIDS 18: 799–804.

19. Pantazis N, Morrison C, Amornkul PN, Lewden C, Salata RA, et al. (2012) Differences in HIV natural history among African and non-African seroconverters in Europe and seroconverters in sub-Saharan Africa. PLoS One 7: e32369.

20. Pantazis N, Morrison C, Amornkul PN, Lewden C, Salata RA, et al. (2012) Differences in HIV natural history among African and non-African seroconverters in Europe and seroconverters in sub-Saharan Africa. PLoS One 7: e32369.

21. Ribeiro RM, Qin L, Chavez LL, Li D, Self SG, et al. (2010) Estimation of the initial viral growth rate and basic reproductive number during acute HIV-1 infection. J Virol 84: 6096–6102.

22. Pilcher CD, Price MA, Hoffman IF, Galvin S, Martinson FE, et al. (2004) Frequent detection of acute primary HIV infection in men in Malawi. AIDS 18: 517–524.

23. Rizzardi GP, De Boer RJ, Hoover S, Tambussi G, Chapuis A, et al. (2000) Predicting the duration of antiviral treatment needed to suppress plasma HIV-1 RNA. J Clin Invest 105: 777–782.

24. Hammer SM, Eron JJ, Reiss P, Schooley RT, Thompson MA, et al. (2008) Antiretroviral treatment of adult HIV infection: 2008 recommendations of the International AIDS Society-USA panel. JAMA 300: 555–570.

25. Dioulasso B, Faso B, Meda N, Fao P, Ky-Zerbo O, et al. (2012) Maternal HIV-1 disease progression 18-24 months postdelivery according to antiretroviral prophylaxis regimen (triple-antiretroviral prophylaxis during pregnancy and breastfeeding vs zidovudine/single-dose nevirapine prophylaxis): The Kesho Bora randomized. Clin Infect Dis 55: 449–460.

26. Chung MH, Kiarie JN, Richardson BA, Lehman DA, Overbaugh J, et al. (2008) Highly active antiretroviral therapy versus zidovudine/nevirapine effects on early breast milk HIV type-1 Rna: a phase II randomized clinical trial. Antivir Ther (Lond) 13: 799–807.

27. Fairall LR, Bachmann MO, Louwagie GM, van Vuuren C, Chikobvu P, et al. (2008) Effectiveness of antiretroviral treatment in a South African program: a cohort study. Arch Intern Med 168: 86–93.

28. Bussmann H, Wester CW, Ndwapi N, Grundmann N, Gaolathe T, et al. (2008) Five-year outcomes of initial patients treated in Botswana’s National Antiretroviral Treatment Program. AIDS 22: 2303–2311.

29. Hughes JP, Baeten JM, Lingappa JR, Magaret AS, Wald A, et al. (2012) Determinants of per-coital-act HIV-1 infectivity among African HIV-1-serodiscordant couples. J Infect Dis 205: 358–365.

30. Mugo NR, Heffron R, Donnell D, Wald A, Were EO, et al. (2011) Increased risk of HIV-1 transmission in pregnancy: a prospective study among African HIV-1 serodiscordant couples. AIDS 25: 1887–1895.

31. Plazy M, Dray-Spira R, Orne-Gliemann J, Dabis F, Newell M-L (2014) Continuum in HIV care from entry to ART initiation in rural KwaZulu-Natal, South Africa. Trop Med Int Health 19: 680–689. doi:10.1111/tmi.12301.

32. Barnabas R, Van Rooyen H, Tumwesigye E, Krows M, Murnane P, et al. (2014) Community HIV testing and linkage to care reduces population viral load in South Africa and Uganda. Conference On Retroviruses And Opportunistic Infections, Boston, USA (Abstract 148).

33. Geng EH, Bwana MB, Muyindike W, Glidden D V, Bangsberg DR, et al. (2013) Failure to initiate antiretroviral therapy, loss to follow-up and mortality among HIV-infected patients during the pre-ART period in Uganda. J Acquir Immune Defic Syndr 63: 64–71.

34. Mugisha V, Teasdale CA, Wang C, Lahuerta M, Nuwagaba-Biribonwoha H, et al. (2014) Determinants of mortality and loss to follow-up among adults enrolled in HIV care services in Rwanda. PLoS One 9: e85774. doi:10.1371/journal.pone.0085774.

35. Jain V, Byonanebye DM, Liegler T, Kwarisiima D, Chamie G, et al. (2014) Changes in population HIV RNA levels in Mbarara, Uganda, during scale-up of HIV antiretroviral therapy access. J Acquir Immune Defic Syndr 65: 327–332.

36. Ruzagira E, Wandiembe S, Abaasa A, Levin J, Bwanika A, et al. (2011) Prevalence and incidence of HIV in a rural community-based HIV vaccine preparedness cohort in Masaka, Uganda. PLoS One 6: e20684.

37. Tanser F, Barnighausen T, Grapsa E, Zaidi J, Newell ML (2013) High coverage of ART associated with decline in risk of HIV acquisition in rural KwaZulu-Natal, South Africa. Science (80- ) 339: 966–971.

S1 Figure: Proportion of infected individuals who are virally suppressed (viral count < 100 counts/ml) at the end of the ten-year simulation period in Uganda (top row) and South Africa (bottom row). Blue, orange and green bars show Options A, B, and B+, respectively.
